# CRISPR technology incorporating amplification strategies: molecular assays for nucleic acids, proteins, and small molecules†

**DOI:** 10.1039/d0sc06973f

**Published:** 2021-03-02

**Authors:** Wei Feng, Ashley M. Newbigging, Jeffrey Tao, Yiren Cao, Hanyong Peng, Connie Le, Jinjun Wu, Bo Pang, Juan Li, D. Lorne Tyrrell, Hongquan Zhang, X. Chris Le

**Affiliations:** Division of Analytical and Environmental Toxicology, Department of Laboratory Medicine and Pathology, Faculty of Medicine and Dentistry, University of Alberta Edmonton Alberta T6G 2G3 Canada xc.le@ualberta.ca hongquan@ualberta.ca +1-780-492-7800 +1-780-492-6416; Li Ka Shing Institute of Virology, Department of Medical Microbiology and Immunology, Faculty of Medicine and Dentistry, University of Alberta Edmonton Alberta T6G 2E1 Canada; School of Public Health, Jilin University 1163 Xinmin Street Changchun Jilin 130021 China

## Abstract

Clustered Regularly Interspaced Short Palindromic Repeats (CRISPR) and CRISPR-associated (Cas) protein systems have transformed the field of genome editing and transcriptional modulation. Progress in CRISPR–Cas technology has also advanced molecular detection of diverse targets, ranging from nucleic acids to proteins. Incorporating CRISPR–Cas systems with various nucleic acid amplification strategies enables the generation of amplified detection signals, enrichment of low-abundance molecular targets, improvements in analytical specificity and sensitivity, and development of point-of-care (POC) diagnostic techniques. These systems take advantage of various Cas proteins for their particular features, including RNA-guided endonuclease activity, sequence-specific recognition, multiple turnover *trans*-cleavage activity of Cas12 and Cas13, and unwinding and nicking ability of Cas9. Integrating a CRISPR–Cas system after nucleic acid amplification improves detection specificity due to RNA-guided recognition of specific sequences of amplicons. Incorporating CRISPR–Cas before nucleic acid amplification enables enrichment of rare and low-abundance nucleic acid targets and depletion of unwanted abundant nucleic acids. Unwinding of dsDNA to ssDNA using CRISPR–Cas9 at a moderate temperature facilitates techniques for achieving isothermal exponential amplification of nucleic acids. A combination of CRISPR–Cas systems with functional nucleic acids (FNAs) and molecular translators enables the detection of non-nucleic acid targets, such as proteins, metal ions, and small molecules. Successful integrations of CRISPR technology with nucleic acid amplification techniques result in highly sensitive and rapid detection of SARS-CoV-2, the virus that causes the COVID-19 pandemic.

## Abstract 2

Clustered Regularly Interspaced Short Palindromic Repeats (CRISPR) and CRISPR-associated (Cas) protein systems revolutionize genome engineering and advance analytical chemistry and diagnostic technology.

## Graphical abstract

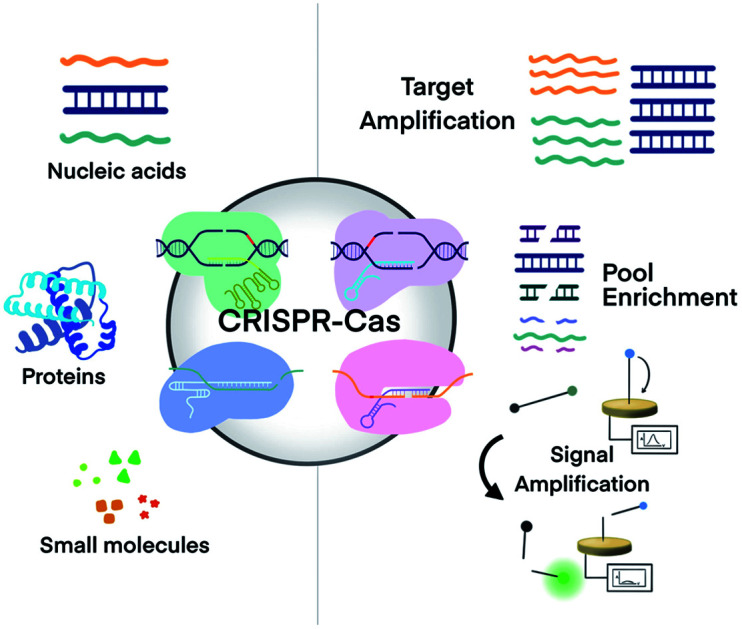

## Introduction

The Clustered Regularly Interspaced Short Palindromic Repeats (CRISPR) and CRISPR-associated (Cas) protein systems have revolutionized our ability to manipulate, regulate, and image genomic nucleic acids.^1–5^ CRISPR–Cas systems use CRISPR RNA (crRNA) or a single-guide RNA (sgRNA) to direct Cas effector proteins to specific nucleic acid sequences for processing, *e.g.*, binding and/or cleavage. Prior to CRISPR–Cas technology, other nucleic acid binding proteins, such as zinc finger nucleases (ZFNs),^6^ transcription activator-like effector nucleases (TALENs),^7^ and meganucleases,^8^ were engineered to bind to and operate on specific genomic loci.^9,10^ Meganucleases, such as LAGLIDADG homing endonuclease, specifically recognize double-stranded DNA sequences of 14 to 40 base pairs and enable modification and deletion of DNA sequences.^8^ ZFNs require multiple zinc-finger motifs to be linked tandemly, with each motif targeting one nucleotide triplet.^10^ TALENs require a DNA-binding domain in which each amino acid binds specifically to one of the four types of nucleotides.^10^ These systems require engineering different fusion proteins for different target sites, and therefore are not widely applicable. CRISPR–Cas technology overcomes this problem. Targeting a different gene sequence can be achieved by using a corresponding crRNA designed to recognize the gene sequence. This programmable feature of the crRNA-mediated guidance of CRISPR is particularly advantageous. Thus, CRISPR–Cas systems have been extensively used to genome editing and transcriptional modulation, and have also been applied to the development of molecular detection and imaging techniques.

Recent research on incorporating CRISPR–Cas systems with various nucleic acid amplification strategies has resulted in the generation of amplified detection signals, improvements in analytical specificity and sensitivity, the enrichment of rare and low-abundance molecular targets, and the development of point-of-care (POC) diagnostic techniques. For example, an amplification reaction using the Cas9 nickase (Cas9nAR) with the help of polymerase and primers can exponentially amplify double-stranded DNA (dsDNA) while avoiding the thermal cycling required by polymerase chain reaction (PCR).^11^ Modular combinations of CRISPR–Cas systems with nucleic acid amplification techniques take advantage of the exponential amplification ability of current nucleic acid techniques and the high specificity of CRISPR–Cas systems.^12–14^ Among the many applications, CRISPR–Cas systems have been incorporated into the development of rapid POC diagnostic tests for the coronavirus disease of 2019 (COVID-19).^15–18^ The potential of CRISPR technology in high-throughput and comprehensive diagnostics of multiple viral infections has recently been demonstrated.^19^ In this review, we highlight how unique features of CRISPR–Cas systems are integrated with innovative nucleic acid amplification techniques, including amplification of the target and the signal, to achieve sensitive and specific detection of nucleic acids, proteins, and small molecules.

## Fundamental features of CRISPR–Cas system

**Fig. 1:**
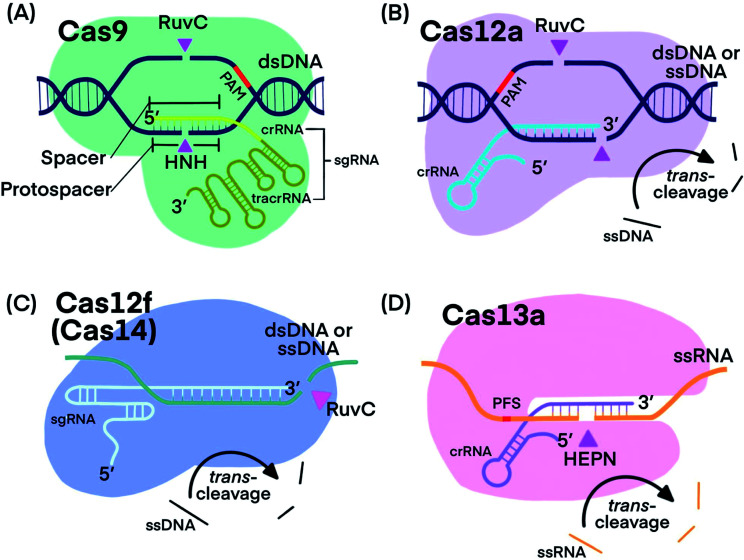
Fundamental components of CRISPR–Cas9, -Cas12a, -Cas12f, and -Cas13a systems. Pink triangles indicate *cis*-cleavage sites.

CRISPR–Cas systems generally function as RNA-guided endonucleases. crRNA guides Cas proteins to specific nucleic acid sequences, where hybridization initiates the nuclease activity of Cas-protein, resulting in nucleic acid cleavage.^1,13,20^ Some systems, such as those that use Cas9, require a *trans*-activating CRISPR RNA (tracrRNA), which binds to crRNA, forming an RNA hybrid.^1^ The crRNA and tracrRNA can be linked into a single guide RNA (sgRNA).^1^ Many CRISPR–Cas systems have been discovered and characterized with each exhibiting distinct cleavage functions and activities, thus making CRISPR technology stand out for diverse applications. Fig. 1 shows the components of the four CRISPR–Cas systems and Table 1 summarizes their unique features that have been used for developing analytical techniques.

**Table 1:** Key features of each CRISPR–Cas system

	Cas9	Cas12	Cas12f (Cas14)	Cas13
Subtypes, homologs, and/or variants discussed in this review	Deactivated Cas 9 (dCas9), Cas 9 nickase (nCas9): Cas9D10A and Cas9H840A	Cas12a (Cpf 1): *Lachnospiraceae bacterium* Cas12a, LbCas12a; Cas12b: *Alicyclobacillus acidiphilus* Cas12b, AapCas12b, *Alicyclobacillus acidoterrestris* Cas12b, AacCas12b	Cas14a	Cas13a (C2c2): *Leptotrichia buccalis* Cas13a, LbuCas13a, *Leptotrichia wadei* Cas13a, LwaCas13a; Cas13b
Size (according to Uniprot)	∼1400 amino acids (aa) (*Streptococcus pyogenes* Cas 9, SpCas9)	∼1300 aa (LbCas12a)	400–700 aa	∼1160 aa (LbuCas13a)
Target (activator)	dsDNA,^1^ ssDNA and ssRNA if PAMmer is provided^28,29^	ssDNA, dsDNA^13^	ssDNA, dsDNA^34^	ssRNA^12,46^
Commonly used spacer length	20 nucleotide (nt)^47^	20 nt^13^	20 nt^39^	28 nt^12^
*Cis*-cleavage products of dsDNA	Blunt ends or PAM-distal 5′ overhang^1,26^	For dsDNA, sticky end contains a 5–7 nt overhang^13^	Sticky end^34^	Not applicable
Specificity	The 6–8 nt near PAM are more specific than other locations;^47^ mismatch in PAM region significantly impairs the activity of SpCas9.^1^	For dsDNA, 4–6 nt at the 3′ end of crRNA is less specific than other locations;^48^ cannot achieve single nucleotide specificity for ssDNA target^39^	Seed region is more specific than other locations in sgRNA^39^	The use of a synthetic mismatch in crRNA results in a single-nucleotide specificity^12^
*Trans*-cleavage substrates	Not applicable	ssDNA^13^	ssDNA^39^	ssRNA^20,46^

Cas9 is well studied for its programmable endonuclease activity.^1,21–24^ The sgRNA of CRISPR–Cas9 systems contains a hairpin-rich region that binds to Cas9 and a 20-nucleotide “spacer” region that binds with the complementary “protospacer” region in the target strand of a dsDNA duplex. Binding between the sgRNA and the DNA target brings Cas9 into close proximity to the target (Fig. 1A). The His-Asn-His (HNH) domain of Cas9 cleaves the strand that is complementary to sgRNA (target strand) and the RuvC domain of Cas9 cleaves the other strand of the dsDNA (non-target strand).^25^ The combined endonuclease activities of the HNH and RuvC domains achieve the cleavage of both strands of the dsDNA of interest, leaving behind two blunt ends or a “protospacer adjacent motif” (PAM)-distal 5′ overhang.^26^ Cas9 can target innumerable DNA sequences; the only requirement is the presence of a “protospacer adjacent motif” (PAM) located 3–4 nucleotides (nt) downstream from the protospacer.^2,27^ Unlike restriction endonucleases, CRISPR–Cas9 systems cleave the specific sites that are pre-determined by specific sgRNA sequences. CRISPR–Cas9 systems can also be programmed to target single-stranded DNA (ssDNA) or single-stranded RNA (ssRNA) by introducing a PAM-presenting oligonucleotides (PAMmer) sequence. A PAMmer is typically designed to hybridize with the target strand and form a pseudo-PAM region to activate Cas9.^28,29^

When a single amino acid in either the RuvC (Cas9D10A) or HNH (Cas9H840A) domain is mutated, Cas9 behaves as a nickase (nCas9); in the first case, it nicks only the target strand of a dsDNA duplex and in the second case, it nicks only the non-target strand.^1,30,31^ Mutating both RuvC and HNH domains produces deactivated Cas9 (dCas9), which is still guided by sgRNA to bind to nucleic acid targets, but does not cleave them.^32^

Cas12, including the well-known subtypes Cas12a and Cas12f (previously referred to as Cas14),^33,34^ lacks the HNH domain, but is still able to achieve PAM-dependent cleavage of dsDNA with its RuvC domain alone (Fig. 1B).^13,35,36^ Cleavage of both the target strand and its complementary non-target strand by Cas12a produces 5–7 nt overhangs because the cleavage sites on the two strands are staggered. Cas12 also targets and cleaves ssDNA, but does not require a PAM recognition^35^ (Fig. 1C). Distinctly, Cas12 cleaves not only the target DNA strand (*cis*-cleavage), Cas12 also exhibits *trans*-cleavage activity (also referred to as collateral cleavage), which cleaves ssDNA indiscriminately.^13,37–39^ Another important feature of Cas12 is its multiple-turnover nuclease activity, which can be harnessed for the amplified detection of nucleic acid targets, including products of other nucleic acid amplification techniques. Although less is known about Cas12f, existing evidence has shown two promising features: Cas12f has a smaller size compared to Cas9 and other Cas12 subtypes (Table 1), making Cas12f useful for imaging applications within cells. Cas12f also has better specificity toward ssDNA activation than Cas12a; thus Cas12f has been used to distinguish single nucleotide differences at certain protospacer sites.^39^

Compared to recombinases and nucleases (*e.g.*, nicking and restriction endonucleases) that have been used in nucleic acid amplification techniques, CRISPR–Cas systems have distinct advantages for improving nucleic acid detection and molecular diagnostics. Both Cas9 and Cas12 enable unwinding of specific dsDNA without the need for single-stranded binding proteins (SSB) that are otherwise required for unwinding dsDNA when recombinases are used. This unwinding ability of Cas9 and Cas12 facilitates the imaging of specific dsDNA sequences in living cells^40,41^ and the development of new fluorescence *in situ* hybridization (FISH) techniques for localized detection of nucleic acids.^42^ The CRISPR–Cas9 and -Cas12 systems allow cleavage of both strands or single strand of different dsDNA sequences by simply altering crRNA, whereas nicking and restriction endonucleases only work on dsDNA sequences containing or adjacent to specific recognition sites. Consequently, the CRISPR–Cas9 and -Cas12 systems can potentially replace nicking or restriction endonucleases for nucleic acid amplification with improved performance.

CRISPR–Cas13 systems target and cleave ssRNA. After Cas13 binds to a target ssRNA, its two domains, called HEPN (higher eukaryotes and prokaryotes nucleotide-binding) domains, are brought together through a conformational change to initiate *cis*-and *trans*-cleavage activities^43–45^ (Fig. 1D). Whereas Cas9 requires PAM, Cas13a and Cas13b proteins prefer a specific nucleotide next to the 3′ end of the protospacer, called the protospacer flanking site (PFS).^20,44^ Cas13 cannot be used as a nicking enzyme because the cleavage site of Cas13 on its target is not fixed and multiple sites on one target can be cleaved.^49^ CRISPR–Cas13 systems exhibit *trans*-cleavage activity that is activated by a specific target, and thus have been widely used as molecular switches.

Although most Cas12 and Cas13 systems can tolerate mismatches between spacer and target strands, crRNA or gRNA can be modified to improve the specificity of CRISPR-based detection. For example, a single nucleotide specificity has been achieved for nucleic acid detection by introducing a synthetic mismatch between crRNA and target.^12,37^ However, the activity and specificity of Cas systems vary among different originating species and target sites. Systematic optimization of crRNA may be required when an existing technique is applied to different targets.

## Using the *trans*-cleavage activity of CRISPR–Cas to generate amplified detection signals

The *trans*-cleavage activity of Cas12 and Cas13 has been used to develop on-off switches that enable amplified detection of nucleic acids. These systems typically function through two essential components: a ribonucleoprotein (RNP) complex between a crRNA and the Cas protein and a nucleic acid signalling reporter. The crRNA in the RNP is designed to hybridize to a nucleic acid target of interest. Altering the spacer domain of the crRNA allows for the flexibility to detect different nucleic acid targets. Hybridization of the crRNA with the nucleic acid target activates the *trans*-cleavage activity of the Cas protein, leading to the cleavage of multiple reporter molecules. Therefore, signal amplification is achieved for sensitive detection of nucleic acid targets. The Cas12-based system can detect both dsDNA and ssDNA targets, whereas the Cas13-mediated system detects RNA targets. Incorporation of established signal generation approaches, such as fluorescence and electrochemical, into nucleic acid reporters enables various options of sensitive readout.

**Fig. 2:**
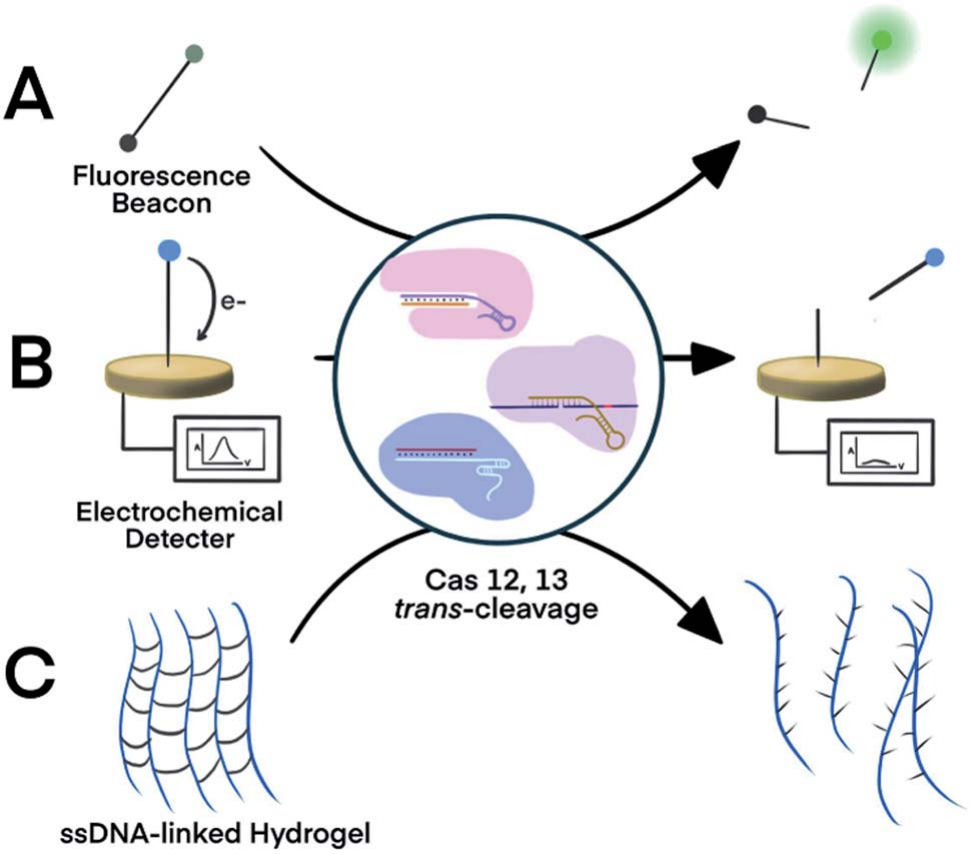
Three representative nucleic acid detection platforms using the *trans*-cleavage activity of CRISPR–Cas12a and -Cas13a. *Trans*-cleavage of ssDNA or ssRNA linker built into (A) a fluorophore and quencher pair,^50,51^ (B) a redox-reactive molecule and electrode,^53,54^ or (C) hydrogel.^55^ Adapted from ref. 50 and 54. Copyright 2017 Wiley and Sons.

The strategy of Cas-mediated generation and amplification of fluorescence^49^ has been used to detect microRNA^50^ and *N*^1^-methyladenosine.^51^ Typically, ssDNA or ssRNA reporters were designed to serve as non-specific substrates for Cas12 and Cas13.^49^ With a fluorophore and a quencher on each end of the short ssDNA or ssRNA, the fluorescence of these reporters was quenched due to fluorescence energy transfer. When the Cas protein was activated in response to the target binding, it indiscriminately *trans*-cleaves the reporters (substrates), which separated the quencher from the fluorophore and restored the fluorescence (Fig. 2A). Cas12 and Cas13 repeatedly cleaved multiple reporter molecules, and therefore amplified the fluorescence signal. The kinetics of the cleavage varied among Cas proteins and the reaction conditions. The turnover number of Cas12 was as high as 1250/s, meaning as many as 1250 substrate molecules were cleaved by one Cas enzyme in one second.^13^ The cleavages of reporters by Cas proteins proceeded at moderate temperature (∼37 °C), making CRISPR–Cas systems suitable for point-of-care testing. Such fluorescence assay has also been realized on automated fluorometer-integrated microfluidic chips for point-of-care serological testing of Ebola virus.^52^ Limited by the signal amplification efficiency of CRISPR–Cas alone, this assay required the input RNA concentration to be higher than 5.45 × 10^7^ copies per mL.

For electrochemical detection, redox-reactive molecules served as detection reporters.^53,54^ The redox-reactive molecule was typically conjugated to a ssDNA or ssRNA substrate that was tethered to an electrode surface. Specific nucleic acid binding to the Cas system initiated the *trans*-cleavage of multiple substrate molecules, which released multiple redox-reactive molecules from the electrode surface and disrupted the electron transfer between the electrode and the redox-reactive molecule (Fig. 2B). The amplified electric signals generated by the release of these redox-reactive molecules provided highly sensitive detection of the nucleic acid target. Compared to conventional electrochemical assays, the target-initiated *trans*-cleavage activity of the Cas system improved the specificity of highly sensitive electrochemical detection. As a consequence, incorporation of the CRISPR technology into electrochemical platforms has achieved discrimination of a single-nucleotide difference in specific nucleic acid targets.^53,54^

An alternative platform used an ssDNA-bridged hydrogel as the detection reporter.^55^ The target-initiated *trans*-cleavage activity of Cas12a cleaved multiple ssDNA that linked hydrogel polymers, which altered the structural properties and permeability of the hydrogel (Fig. 2C). Hydrogels disintegrated by the Cas12a-mediated cleavage released cargos, such as fluorophores, for subsequent detection. Other strategies for hydrogel manipulation, such as DNA strand displacement or DNA-initiated conformational change, required a 1 : 1 ratio between target nucleic acids and the ssDNA cross-linkers, thus requiring high concentrations of the target, resulting in suboptimal sensitivity. Target choice was also limited because strand displacement required careful design of oligo sequences. In contrast, the multiple-turnover nature of Cas12a *trans*-cleavage activity allowed a target-binding event to trigger the cleavage of multiple ssDNA hydrogel linkers.^55^ The cleavage of ssDNA cross-linker also changed the permeability of hydrogel, allowing the use of polyacrylamide hydrogel as a fluidic valve on paper-based microfluidic device. Sample with nucleic acid of interest activated Cas12a to cleave ssDNA cross-linkers, which resulted in slow gel formation and thus a high flow rate. Liquid flow-through the hydrogel layer flowed along the channel for electrical detection or mixed with dyes for visual detection. Although *trans*-activating CRISPR–Cas systems alone were not sensitive enough to detect nucleic acid of extremely low abundance, incorporating an isothermal nucleic acid amplification step improved the detection limit of a hydrogel platform from 400 pM dsDNA to 11 aM viral RNA.^55^

Also a visual point-of-care platform, a volumetric Bar–Chart chip used ssDNA to link platinum nanoparticles and magnetic beads. Cas12 recognizing DNA targets released platinum nanoparticles from the immobilized magnetic beads. Free platinum nanoparticles were then transferred to a channel containing H_2_O_2_. The amount of O_2_ generated was visualized by the position of a dye pushed by the generated O_2_.^56^

## Incorporating CRISPR–Cas after nucleic acid amplification to improve the specificity of assays

The *trans*-cleavage activity of Cas12 and Cas13 has been used to detect amplification products (amplicons) of various nucleic acid amplification techniques. Integrating a CRISPR–Cas system after nucleic acid amplification improves the nucleic acid detection and achieves three main benefits. First, CRISPR–Cas systems recognize specific sequences of amplicons and differentiate the specific amplicons from byproducts of amplification reactions, thereby improving the specificity. Second, the multiple turnover *trans*-cleavage activity of Cas12 and Cas13 leads to repeated cleavage of nucleic acid signaling reporters, generating amplified readout signals for detection, thus improving sensitivity. Third, CRISPR–Cas systems facilitate the generation of diverse readout signals, broadening applicability.

**Fig. 3:**
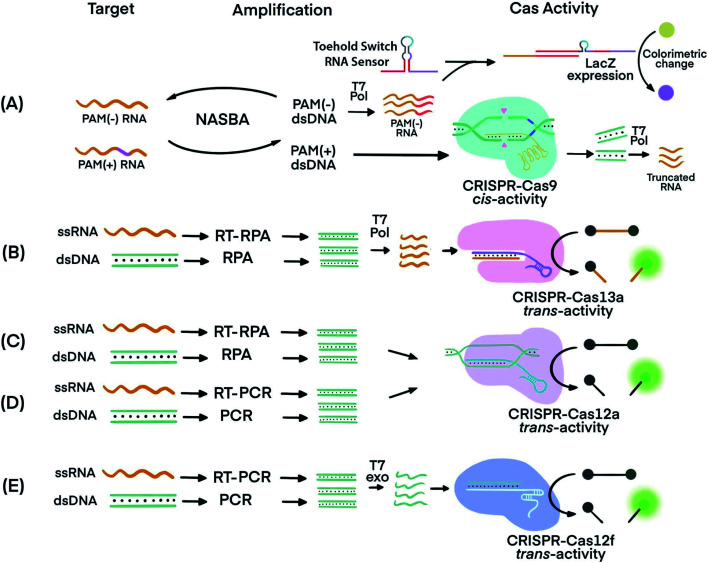
Techniques that use CRISPR–Cas systems to generate amplified signals from amplicons produced by nucleic acid amplification techniques. (A) Nucleic Acid Sequence-Based Amplification CRISPR Cleavage (NASBA-CC) followed by CRISPR–Cas9. A difference in the presence or absence of a PAM sequence in the viral RNA was used to differentiate the viral strains.^58^ (B) Specific High-sensitivity Enzymatic Reporter unLOCKing (SHERLOCK) assay incorporating recombinase polymerase amplification (RPA) with Cas13a.^12^ T7 RNA polymerase was necessary before the CRISPR–Cas13a reaction. (C) DNA Endonuclease-Targeted CRISPR Trans Reporter (DETECTR) assay incorporating RPA with CRISPR–Cas12a.^13^ (D) One HOur Low-cost Multipurpose highly Efficient System (HOLMES) incorporating PCR amplification with CRISPR–Cas12a *trans*-cleavage activity.^61^ (E) RT-PCR or PCR incorporated with CRISPR–Cas12f (Cas14a).^39^ Pol: polymerase. RT: reverse transcription. T7 Pol: T7 polymerase. T7 exo: T7 exonuclease. Adapted from ref. 58. Copyright 2016 Elsevier.

The recognition of specific sequences by CRISPR–Cas acts as a confirmation that the intended sequences were amplified and detected. In one example, Nucleic Acid Sequence-Based Amplification (NASBA)^57^ was combined with CRISPR–Cas9, resulting in a NASBA-CRISPR cleavage assay. The requirement of Cas9 for its target sequence to contain a PAM region was used to distinguish between American and African strains of Zika virus (ZIKV) (Fig. 3A).^58^ After amplification using NASBA, the amplicons of American ZIKV contained a PAM region and permitted Cas9-mediated cleavage, while the amplicons of the African ZIKV without PAM remained uncut. The intact amplicon of the African ZIKV interacted with a subsequent sensing system to induce an observable color change. Thus, two strains differing by one nucleotide in the PAM region were differentiated.

Cas proteins with switchable *trans*-cleavage activity can be used to generate amplified signals after isothermal amplification of a specific target molecule. This strategy is exemplified in SHERLOCK (Specific High-Sensitivity Enzymatic Reporter UnLOCKing), facilitated by CRISPR–Cas13. Target DNA or RNA was amplified by recombinase polymerase amplification (RPA) or reverse transcription (RT) RPA,^59^ respectively. RPA was chosen because it is highly sensitive, rapid, and has an operation temperature (37–42 °C) compatible with that of Cas13 (∼37 °C). After RPA, amplicons were transcribed into ssRNA by T7 polymerase. The amplified ssRNA product initiated CRISPR–Cas13a *trans*-cleavage activity and generated an amplified fluorescence signal for detection (Fig. 3B).^12^ A sample preparation technique, known as Heat Unextracted Diagnostic Samples to Obliterate Nucleases (HUDSON), has been developed to be paired with SHERLOCK.^14^ The HUDSON–SHERLOCK assay was able to detect Zika and Dengue viruses in patients' urine and saliva samples at an equivalent nucleic acid concentration as low as 1 copy per μL. Multiple nucleic acids have also been analyzed simultaneously using the SHERLOCK platform by taking advantage of the preferential cleavage activity of Cas13a and Cas13b orthologs for various dinucleotide motifs at their respective cleavage sites.^60^ The different CRISPR–Cas13 orthologs could then be paired with pre-designed reporters. The reporters consisted of each of the orthologs' preferred dinucleotide motifs and different fluorophore-quencher pairs. For example, in the same reaction solution LwaCas13a cleaved reporters for detecting Zika virus, which consisted of an adenine-uracil site and TEX red fluorescence dye, and CcaCas13b cleaved reporters containing a uracil-adenine site and Cy5 far-red fluorescence dye for the detection of Dengue virus.

A similar strategy to SHERLOCK was developed, except using CRISPR–Cas12 instead of CRISPR–Cas13. Because Cas12 targets DNA, the DNA amplicons can be directly detected without the need for additional transcription (Fig. 3C). An RPA-Cas12a detection platform, termed DNA Endonuclease-Targeted CRISPR Trans Reporter (DETECTR), was able to identify viral strains from DNA extracted from patient samples.^13^ The temperature compatibility of RPA and CRISPR–Cas12 makes it possible for both processes to take place in the same reaction tube, a feature useful for developing simple POC tests.

A DETECTR technique using the Cas14a (Cas12f) protein was able to discriminate single nucleotide polymorphisms (SNPs) in HERC2, a gene involved in eye color^39^ (Fig. 3E). The CRISPR–Cas14a (Cas12f) system requires full complementarity in the seed region of sgRNA, a feature considered for achieving single nucleotide specificity. The SNPs of the HERC2 gene residing in the seed region in the middle of the protospacer, as well as SNPs within the PAM region, completely deactivated Cas14a.^39^ Due to the single nucleotide specificity in the seed region, the Cas14a-DETECTR technique successfully distinguished the SNP corresponding to blue eyes from that of brown eyes.

Other nucleic acid amplification techniques have also been paired with CRISPR–Cas systems. For example, a technique called one-HOur Low-cost Multipurpose highly Efficient System (HOLMES) used PCR to amplify target nucleic acids and detected the amplified products using a CRISPR–Cas12a system (Fig. 3D).^61^ HOLMES version 2 detected amplicons from PCR and loop-mediated isothermal amplification (LAMP). However, because the nucleic acid target did not have a suitable PAM region nearby, HOLMES version 2 used two strategies. First, because ssDNA activation of Cas12 is PAM independent, asymmetric PCR was used to generate ssDNA amplicons to avoid the requirement of PAM. Second, since amplicons from LAMP were dsDNA, PAM sequences were introduced through careful design of primers. Both ssDNA and dsDNA amplicons initiated the *trans*-cleavage activity of Cas12a for detection. The PCR amplification and CRISPR–Cas12a detection steps must be conducted separately because the CRISPR–Cas12a system is inactive at the denaturation temperature (95 °C) used during PCR. Typical reaction temperatures for LAMP and RT-LAMP (60–65 °C)^62^ could be tolerated by Cas12b (>40 °C). Therefore, incorporating LAMP or RT-LAMP with CRISPR–Cas12b has enabled isothermal amplification and signal generation to occur in one procedural step.^63^

SHERLOCK and DETECTR are being used to develop potential POC tests to address the diagnostic needs of the COVID-19 pandemic.^15,64^ Current molecular diagnosis of COVID-19 is mainly based on reverse transcription (transcriptase) quantitative polymerase chain reaction (RT-qPCR) to detect viral RNA of Severe Acute Respiratory Syndrome – Coronavirus 2 (SARS-CoV-2).^65–67^ Diagnostic challenges include time-consuming pre-analytical and analytical processes and the requirement for well-equipped laboratories with trained personnel to conduct RT-qPCR.^16^ Broughton *et al.*^15^ developed SARS-CoV-2 DETECTR, using RT-LAMP and CRISPR–Cas12, to detect the virus's genes encoding the envelope (E) and nucleocapsid (N) proteins. The authors reported a 100% clinical specificity from the analysis of RNA of clinical samples. Using RT-RPA and Cas13, Zhang *et al.*^64^ developed a SHERLOCK lateral flow technique, which was able to detect 10–100 copies of SARS-CoV-2 genes per μL in under one hour. Both DETECTR and SHERLOCK techniques require only heating blocks or water baths, lateral flow dipsticks, a microcentrifuge (for SHERLOCK), pipettes, and pipette tips, which are all portable and amenable to POC testing. An improved version of SHERLOCK screening test for COVID-19, SHERLOCK Testing in One Pot (STOP), integrated sample processing, nucleic acid amplification, and detection in one-pot.^17^ Joung *et al.*^17^ released viral RNA from nasopharyngeal swabs by incubating the swabs with QuickExtract reagents for 10 min at room temperature or 60 °C. The released RNA was captured on magnetic beads and amplified using RT-LAMP; and the amplicon was detected using CRISPR–Cas12b. This one-pot assay achieved the detection of 100 copies of SARS-CoV-2 RNA within 1 h. Analysis of 202 SARS-CoV-2 positive samples and 200 SARS-CoV-2 negative samples demonstrated a clinical sensitivity of 93.1% and a clinical specificity of 98.5%. Key to the success of this “one-pot” assay was the use of a new Cas ortholog, AapCas12b, because this enzyme (from *Alicyclobacillus acidiphilus*) remained active at the temperature of RT-LAMP reactions (60 °C). Previously used, commercially available Cas12a operates at a lower temperature (*e.g.*, 25–40 °C), which is incompatible with temperature conditions of RT-LAMP.

**Fig. 4:**
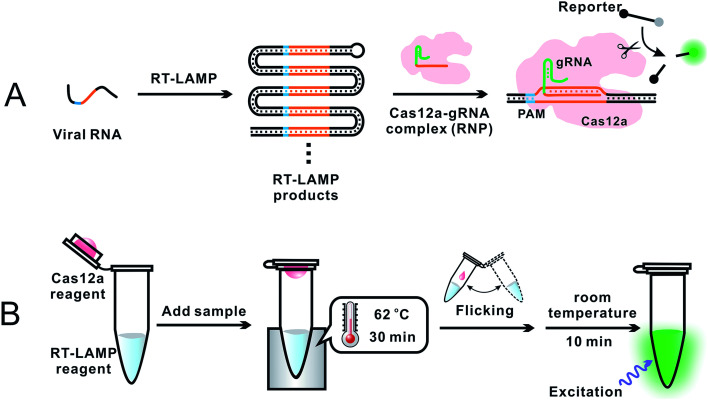
Schematics showing (A) general principle and (B) overall operation of an assay incorporating RT-LAMP with CRISPR–Cas12a for the detection of SARS-CoV-2.^18^ (A) Specific E and N gene sequences of SARS-CoV-2 RNA were amplified using RT-LAMP. The RT-LAMP products were scanned by the Cas12a–gRNA ribonucleoprotein (RNP) complex. RNP recognized the specific sequence complementary to gRNA, activating the *trans*-cleavage activity of Cas12a. The active Cas12a system cleaved a short ssDNA reporter (8 nt) that was labeled with a pair of fluorophore and quencher. The cleavage of the reporter separated the quencher from the fluorophore, resulting in the generation of fluorescence. (B) A 0.2 mL PCR tube contained the RT-LAMP reagent mixture (25 μL or lyophilized) at the bottom and the CRISPR–Cas12a detection reagent mixture (10 μL liquid or lyophilized) inside the cap. A sample was added into the bottom of the tube and mixed with the RT-LAMP reagents. The tube was placed in a heating block and the bottom of the tube was maintained at 62 °C for 30 min to allow for RT-LAMP reactions while the temperature in the cap was about 31 °C. The tube was then removed from the heating block, and the subsequent procedures were performed at room temperature. Inverting and wrist-flicking of the tube forced the Cas12a reagents to mix with the RT-LAMP amplicons in the bottom. Green fluorescence was generated at room temperature and was visualized under the excitation of a handheld UV lamp. Reproduced with permission from ref. 18. Copyright 2020 American Chemical Society.

To overcome the problem of incompatible temperature conditions between RT-LAMP and CRISPR–Cas12a, Pang *et al.*^18^ placed RT-LAMP and CRISPR–Cas12a reagents separately in the same reaction tube (0.2 mL PCR tube): RT-LAMP reagents on the bottom and CRISPR–Cas12a reagents on the cap of the tube (Fig. 4). While the bottom of the tube was heated to 62 °C, using a heating block, to allow for optimum RT-LAMP reaction, the temperature on the cap was about 31 °C. After a 30 min RT-LAMP amplification, the tube was removed from the heating block. The tube was inverted and flicked to mix the amplicon with the CRISPR–Cas12a detection reagents at room temperature. Fluorescence was generated immediately and ready for detection within 10 min. The sequence-specific recognition of the amplicon by the gRNA and Cas12a significantly enhanced the detection specificity. Analysis of RNA extracts of 100 respiratory swab samples (equal number of SARS-CoV-2 positive and negative) showed a clinical specificity of 100% and clinical sensitivity of 94%. This assay required a single controlled temperature for RT-LAMP amplification. The integration of isothermal amplification with the CRISPR–Cas12a detection in a single tube simplified operation and minimized the risk of cross-contamination during the assay.

RT-RPA has also been combined with CRISPR for the detection of SARS-CoV-2.^64,68^ The operation temperature of RT-RPA (37–42 °C) is similar to that of Cas12a (∼37 °C). Thus, RT-RPA isothermal amplification and CRISPR–Cas12a detection are considered compatible in principle. In practice, however, it is challenging to achieve reproducible detection of SARS-CoV-2 in a single tube. Zhang and colleagues pointed out that “while we have focused on recombinase polymerase amplification in the past,^12,14,60^ LAMP reagents are readily available from multiple commercial suppliers, are easily multiplexed,^59^ and rely on defined buffers that are amenable to optimization with Cas enzymes”.^17^

## Incorporating CRISPR–Cas before nucleic acid amplification to enrich for rare and low-abundance targets

Incorporating CRISPR–Cas before nucleic acid amplification has achieved two main objectives: the enrichment of rare and low-abundance nucleic acid targets and depletion of abundant interfering nucleic acids. The challenge is exemplified by the need for detecting tumor-specific somatic mutations in the circulating tumor DNA (ctDNA) from patients' blood that contains several orders of magnitude higher amounts of wild-type DNA sequences.^69^ Two strategies have been developed. The first used the sequence-specific binding ability of dCas9 to enrich the target of extremely low abundance, and the second harnessed the PAM-dependent cleavage by Cas9 to deplete unwanted high-abundance species. The ability of CRISPR–dCas9 and dCas12 to specifically recognize and bind to dsDNA and ssDNA without the need of denaturation enabled enrichment of rare and low-abundance nucleic acid targets, whereas the sequence-specific cleavage of CRISPR–Cas9 and Cas12 was used for depletion of abundant interfering nucleic acids.

**Fig. 5:**
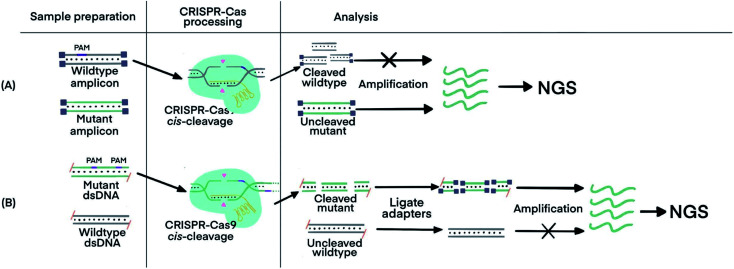
CRISPR–Cas techniques to enrich specific nucleic acids of low abundance for subsequent analysis. (A) Enrichment of target sequences by depletion of wildtype nucleic acids using CRISPR–Cas9 cleavage before target amplification for Next Generation Sequencing (NGS) using Depletion of Abundant Sequences by Hybridization (DASH).^70^ (B) Enrichment of target sequences by amplifying target fragments after CRISPR–Cas9 cleavage using Finding Low Abundant Sequences by Hybridization (FLASH).^71^ The absence of 5′ phosphate groups are indicated by the hyphens and the ligated adapters are indicated by the small purple squares. Adapted from ref. 70. Copyright 2016 Springer Nature. Adapted from ref. 71. Copyright 2019, Oxford University Press.

CRISPR–Cas9 was designed to target and cleave naturally occurring sequences containing PAM regions, leaving sequences with mutated PAM regions uncleaved. Subsequent amplification of the uncleaved mutant sequence resulted in its enrichment. A technique called Depletion of Abundant Sequences by Hybridization (DASH) was able to deplete wild-type mitochondrial ribosomal RNA (rRNA) extracted from cerebrospinal fluid samples and to enrich mutant rRNA (Fig. 5A).^70^ Another technique, called CRISPR-mediated Ultrasensitive Detection of target DNA (CUT-PCR), depleted abundant nucleic acids using sgRNA complementary to wild-type sequences for cleavage by Cas9. The uncleaved oncogenic sequences of circulating tumor DNA from patient blood samples were enriched by subsequent PCR amplification.^69^

CRISPR–Cas9 has also been used to target and cleave sequences of interest, resulting in shorter oligos for subsequent enrichment *via* amplification. This technique, called Finding Low Abundant Sequences by Hybridization (FLASH), has been used to enrich target fragments for next-generation sequencing (NGS) (Fig. 5B).^71^ Phosphate groups were removed from the ends of all genomic DNA or cDNA to prevent the ligation of sequencing adapters that are required for NGS. Cas9 complexes with tracrRNA and crRNA complementary to the targeted sequences produced short oligos from only the mutant DNA. The newly generated ends, containing phosphate groups, could then be linked to the adapters for amplification and sequencing.

CRISPR–dCas9 with only sequence-specific binding and no cleavage activity was used as a recognition probe to extract and separate nucleic acids of interest from complex samples. Typically, the dCas9 protein was conjugated with a tag (*e.g.*, histidine) recognizable by antibodies coated on magnetic beads. Complexes of dCas9-sgRNA ribonucleoprotein (RNP) with the target nucleic acid could be captured on the magnetic beads and separated under a magnetic field. For example, mutated epidermal growth factor genes from non-small cell lung cancers of patients were enriched using a CRISPR–dCas9 technique and detected using qPCR.^72^ Without this enrichment, qPCR was unable to detect low-abundance mutations. In addition to the enrichment of a single target using the CRISPR–dCas9 technique, the programmable ability to use multiple sgRNAs, along with dCas9, allows for simultaneous capture of multiple targets for multiplexed detection.

## Integrating CRISPR–Cas within amplification strategies to achieve isothermal amplification

The ability of CRISPR–Cas9 systems to unwind dsDNA at a moderate temperature (37 °C) has been used to develop and improve isothermal exponential amplification techniques. Exponential amplification of nucleic acids requires separation of dsDNA to ssDNA. Conventional PCR uses high temperature (*e.g.*, 95 °C) to denature dsDNA for each cycle of amplification. Current isothermal exponential amplification techniques require the use of multiple enzymes and proteins to facilitate unwinding and separation of dsDNA. A special feature of Cas9, including the wild-type Cas9, nCas9, and dCas9, is that they can unwind dsDNA to generate ssDNA at 37 °C. Therefore, CRISPR–Cas systems can replace multiple enzymes used in several isothermal exponential amplification techniques. Commonly used restriction enzymes and nicking endonucleases recognize specific sequences of 4–6 base pairs with limited programmability.^11^

Because Cas9 and nCas9 can unwind dsDNA at 37 °C, primers can hybridize to the unwound strand for subsequent amplification without the initial denaturation of the dsDNA target.^73^ The nuclease activity of CRISPR–Cas9 systems can be programmed using sgRNA to target a broader range of sequences. In addition, the spacer of sgRNA can be designed to target multiple sites of dsDNA.^42^

The sequence recognition and *cis*-cleavage properties of CRISPR–Cas9 enable nicking of long nucleic acid strands at specific sites to produce shorter strands for subsequent amplification.^74,75^ For example, EXPonential Amplification Reaction (EXPAR) is a highly sensitive technique in which short-length ssDNA targets are exponentially amplified.^76,77^ The ssDNA produced by CRISPR–Cas9 served as the primer to initiate EXPAR.^74^ Although EXPAR in combination with CRISPR–Cas9 achieved aM sensitivity, it suffered from poor specificity because CRISPR–Cas9 activation tolerated several mismatches between the spacer and protospacer regions. Only single-base mismatches at the dinucleotide cleavage site could be distinguished when dsDNA was processed.^74^ CRISPR–Cas9 *cis*-cleaved DNA was also used to initiate combinations of amplification methods such as strand displacement amplification (SDA) followed by EXPAR. This combination resulted in Nicking-Triggered EXPAR (NTEXPAR)^75^ (Fig. S1, ESI††). Application of these techniques to long ssRNA has yet to be explored and remains a possibility with the use of PAMmers.^28^

**Fig. 6:**
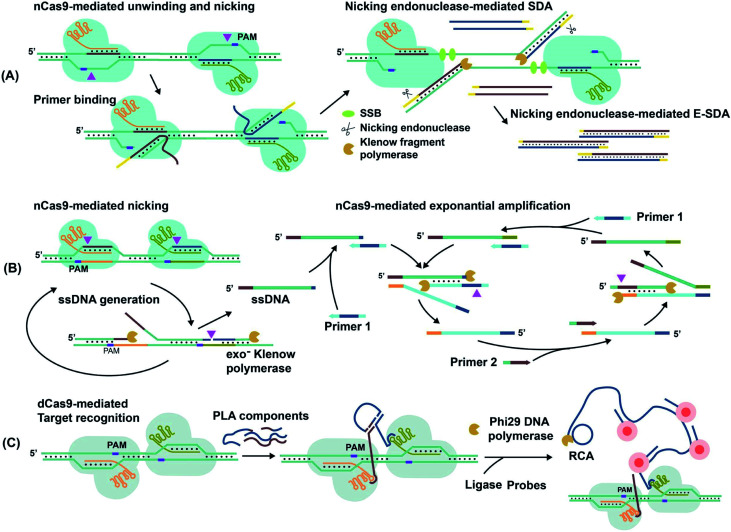
Amplification of target nucleic acids facilitated by CRISPR–Cas9. (A) CRISPR–nCas9 initiates and commences strand displacement amplification (E-SDA).^73^ (B) CRISPR–nCas9 facilitates strand displacement in Cas9n-based amplification reaction (Cas9nAR).^11^ Adapted from ref. 11. Copyright 2019 Wiley-VCH Verlag GmbH & Co. KGaA, Weinheim. (C) The simultaneous binding of two CRISPR–dCas9 probes along a gene locus initiates proximity ligation assays (PLA).^42^ Adapted from ref. 42. Copyright 2012 Royal Society of Chemistry. Adapted from ref. 73. Copyright 2016 Springer Nature.

For techniques such as exponential strand displacement amplification (E-SDA),^78^ an initial 95 °C heating step is required to denature dsDNA into ssDNA for binding of primers. Instead of heat, CRISPR–Cas9-triggered nicking-endonuclease-mediated Strand Displacement Amplification (CRISDA) used two nCas9 proteins to unwind and nick the ends of the dsDNA region of interest (Fig. 6A).^73^ Primers containing nicking endonuclease sites then bind to the 3′ overhang of the nicked strand. To commence E-SDA, the polymerase extended the primers with the assistance of ssDNA binding proteins (SSBs) to stabilize the unwound DNA. The newly formed strand was released after nicking by the endonuclease, facilitating exponential amplification. Although the dsDNA unwinding by nCas9 occurred at a constant temperature, which suggested the potential for single-tube analysis, the authors conducted the analysis in two separate steps. To prevent sgRNAs from being blocked by hybridization to the primers, they used nicking endonuclease in the second step of E-SDA after unwinding by nCas9.

Alternatively, Cas9n-based amplification reaction (Cas9nAR), which used RuvC-mutated nCas9 (D10A), did not require nicking endonucleases (Fig. 6B).^11^ Cas9nAR operated in two circuits. The first circuit used two sgRNAs for CRISPR–Cas9n complexes to nick a targeted length of the dsDNA in two locations. Polymerase extension at the upstream nicking site synthesized a new ssDNA target strand and displaced the original nicked strand. The second nicking facilitated the release of the original nicked strand. This strand (in green color) entered the second circuit which commenced amplification using two primers. Primer 1 first hybridized to the ssDNA and initiated extension, forming a PAM sequence for Cas9n to nick and release another ssDNA (light blue), complementary to the first ssDNA. Primer 2 hybridized to this new ssDNA, facilitating extension, nicking, and release of ssDNA for propagation of the reaction. The combination of two primers and two CRISPR–nCas9 enzymes resulted in the exponential amplification and single-molecule (2 copy/20 μL reaction) detection at 37 °C.

Simultaneous binding events of two sgRNA-dCas9 probes can be used to initiate isothermal amplification techniques. For example, the DNA oligos required for proximity ligation assay (PLA) and subsequent rolling circle amplification (RCA)^79^ were assembled by proximal sgRNA-dCas9 binding events (Fig. 6C).^42^ PLA oligos were hybridized to each stem-loop region of the bound sgRNA-dCas9 molecules, where simultaneous binding favored the completion of PLA assembly. Enzymatic DNA ligation then joined PLA components to form a circular template that was amplified by RCA. The amplified product remained attached to the PLA oligos that were anchored to sgRNA–dCas9. Multiple fluorophore-oligos could then bind to the extended product to generate an amplified fluorescence signal at target genomic loci for imaging applications.

## Amplified detection of non-nucleic acid targets with CRISPR–Cas *trans*-activity

CRISPR–Cas systems can be used in combination with functional nucleic acids (FNAs) and molecular translators for the detection of non-nucleic acid targets, including metal ions, proteins, and small molecules.^54,80–82^ FNAs and molecular translators convert the detection of non-nucleic acid targets into surrogate nucleic acid targets, such as ssDNA, which are amenable for initiating amplified detection strategies.^54,80^ DNA aptamers and deoxyribozymes (DNAzymes), which are chemically more stable and easier to handle than their RNA counterparts, have been frequently used.^83–88^ CRISPR–Cas12 has been used for non-nucleic acid target detection because Cas12 targets both dsDNA and ssDNA and has the additional benefit of *trans*-cleavage activity upon target binding.^13^ Molecular translators other than aptamers and DNAzymes exist and can also convert the detection of non-nucleic acid targets into surrogate nucleic acid targets that can be detected by CRISPR–Cas techniques.^81^

**Fig. 7:**
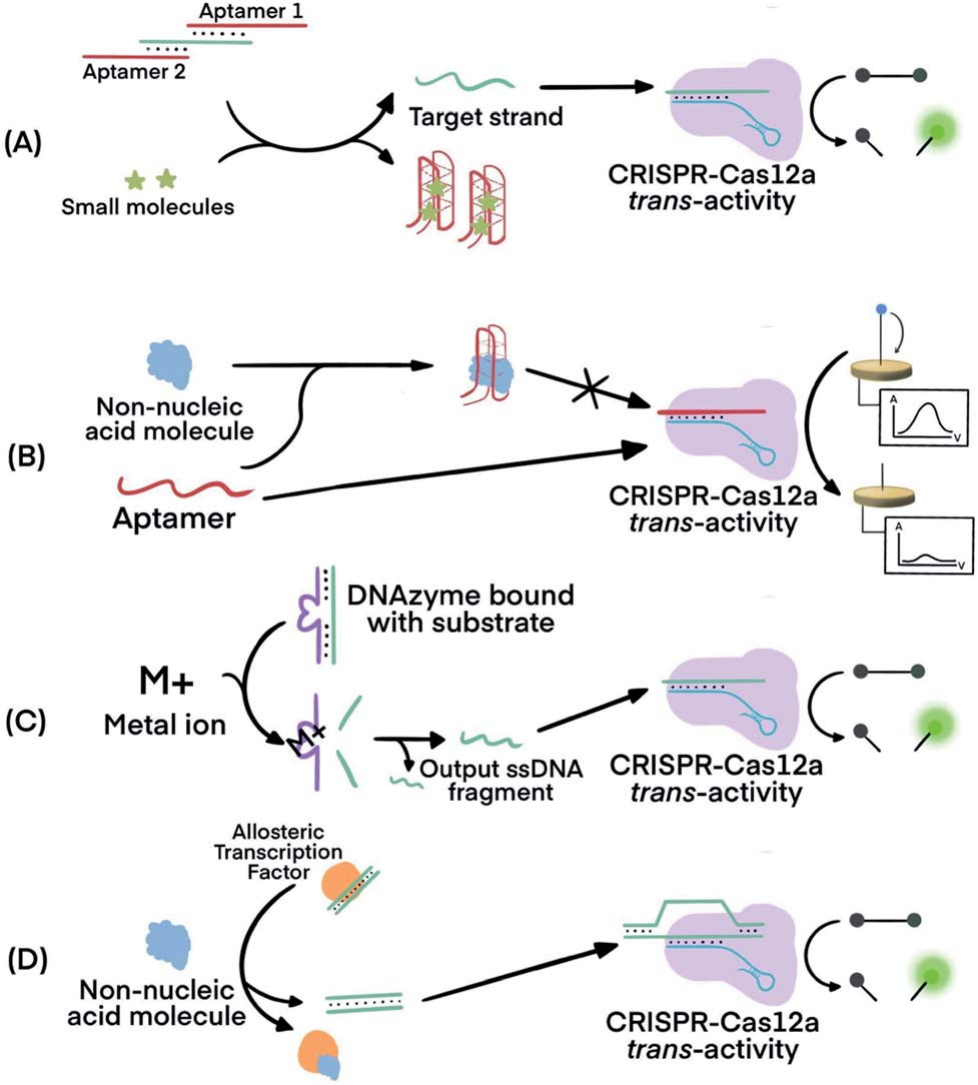
Combining FNAs and molecular translators with CRISPR–Cas technology for the detection of non-nucleic acid targets. (A) The activator, the target ssDNA complementary to crRNA, is locked by two copies of an aptamer. Binding of the aptamer to small molecules (*e.g.*, ATP) releases the activator for hybridization to crRNA and activation of CRISPR–Cas12a *trans*-activity.^80,82^ (B) Binding of the target molecule to its aptamer prevents the activation of CRISPR–Cas12a. The unbound aptamer activates CRISPR–Cas12a.^54^ (C) Metal ions serve as co-factor(s) for an RNA-cleaving DNAzyme to generate output ssDNA for CRISPR–Cas12a activation.^80^ (D) Binding of allosteric transcription factor (aTF) to the target molecule releases output dsDNA for CRISPR–Cas12a activation.^81^ Adapted from ref. 54. Copyright 2019 Wiley-VCH Verlag GmbH & Co. KGaA, Weinheim. Adapted from ref. 80. Copyright 2020 American Chemical Society. Adapted from ref. 81. Copyright 2016 Springer Nature.

In one example, Lu's group used two copies of an adenosine triphosphate (ATP) aptamer to hybridize to an ssDNA (Fig. 7A). The ssDNA was designed to be complementary to crRNA so the sequestering of the ssDNA by the aptamer silenced the activity of Cas12a.^80^ The binding of the aptamers to the ATP target decreased the stability of their hybridization with the ssDNA, resulting in the release of the ssDNA. The released ssDNA could bind to the crRNA and activate the Cas12a for *trans*-cleavage activity to produce amplified detection signals. Alternatively, crRNA was designed to hybridize directly with the aptamer^54^ (Fig. 7B). Folding of the aptamer following its binding to its target displaced its hybridization to the crRNA which turned off the activity of Cas12a. This strategy has been used for the electrochemical detection of TGF-α1 using an aptamer binding to TGF-α1. In the presence of the TGF-α1 target, fewer aptamers were available to bind to crRNA and to activate the *trans*-cleavage activity of CRISPR–Cas12a. The reporter consisted of a ssDNA conjugated to a redox-reactive species, methylene blue, on one end and thiolated on the other end for tethering onto a gold electrode. Lower activation of CRISPR–Cas12a resulted in reduced cleavage of the substrate. Methylene blue in the uncleaved reporter allowed electron exchange with the electrode to generate electrochemical signal.^54^

Metal dependent DNAzymes can cleave their substrates, ssDNA containing RNA cleavage sites, only in the presence of metal ion cofactors. These DNAzyme substrates can be designed to produce ssDNA complementary to crRNA to activate the *trans*-activity of CRISPR–Cas12a for amplified detection of the metal ion (Fig. 7C). In one example, Na^+^-dependent DNAzyme was paired with CRISPR–Cas12a for the detection of Na^+^.^80^ The variety of metal-dependent DNAzymes (*e.g.*, Mg^2+^, Mn^2+^, Pb^2+^, Zn^2+^, Co^2+^, Cu^2+^, and more^89,90^) provides opportunities to develop CRISPR–Cas12a-paired strategies for the selective, sensitive, and multiplex detection of multiple metal ions.

Allosteric transcription factors (aTF), naturally occurring proteins, are capable of binding to small molecules and to particular dsDNA motifs. The affinity of aTFs for the dsDNA motifs changes upon binding to various small molecules. Liang *et al.* used aTFs as molecular translators and developed a CaT-SMelor technique (CRISPR–Cas12a- and aTF-mediated small molecule detector) for the detection of various small molecules.^81^ (Fig. 7D) They designed the sequence of the dsDNA to contain motifs for binding to both the aTF and a PAM sequence. Binding of the aTF to a small-molecule target induced a conformational change of the aTF, resulting in the release of the dsDNA. The released dsDNA, containing PAM, activated CRISPR–Cas12a *trans*-activity to generate detection responses. The authors achieved concentration-dependent, quantitative detection of various small molecules (Fig. S2, ESI††).

Compared to the detection of nucleic acids, the detection of non-nucleic acid targets has an inferior sensitivity, even with the use of FNAs and CRISPR–Cas strategies. The first reason is a lower turnover rate of *trans*-cleavage activity of Cas12a when it is activated by ssDNA than by dsDNA.^13^ Most FNA molecular translators have been designed to generate output ssDNA. Second, DNAzymes display high cleavage activity only with high concentrations of metal ion cofactors (sub-mM to mM).^91,92^ For the detection of trace amounts of metal ions (*e.g.*, heavy metals), DNAzymes may not produce adequate amounts of ssDNA fragments for the activation of CRISPR–Cas12. Third, the efficiency of releasing output ssDNA or dsDNA in response to the target is usually low. For example, the use of aTF as a molecular translator activated Cas12a in response to pM levels of the output dsDNA, while the overall assay was only able to detect nM concentrations of small molecule targets.^81^ Both the binding affinity of the translator to the non-nucleic acid target and the release efficiency of the output DNA greatly affect the sensitivity of these detection strategies.

## Concluding remarks, outlook, and perspectives

CRISPR–Cas systems offer new opportunities for developing diverse analytical techniques and assays. CRISPR–Cas systems can specifically recognize target sequences and have been used for imaging target genes in live and fixed cells.^40–42,93,94^ The feature of recognizing target sequences has also been incorporated into various types of detection methods, such as electrochemical detection, lateral flow assays, nanomaterial-based sensors, and nano/microfluidics.^5,56,95,96^ For example, dCas9 was immobilized on graphene-based microchips for the detection of target genes.^95^ Incorporating CRISPR technology with nucleic acid amplification strategies represents another important research advance for CRISPR-based analytics because the coupling can lead to substantial improvements in sensitivity, specificity, and other analytical characteristics.

The *trans*-cleavage activity of CRISPR–Cas systems improves detection sensitivity of nucleic acids techniques. Hybridization of the crRNA with the nucleic acid target activates the *trans*-cleavage activity of the Cas protein, leading to the cleavage of multiple reporter molecules. Therefore, signal amplification is achieved for sensitive detection of nucleic acid targets. The *trans*-cleavage activity varies among Cas types and homologs. For a specific Cas protein, its *trans*-cleavage activity is also related to sequences of the target sites. The reported turnover number of LbCas12a is five times higher with dsDNA as the target (1250/s) than with ssDNA as the target (250/s).^13^ Large discrepancies exist in literature among the reported *trans*-cleavage rates for a specific Cas protein, which may be due to differences in methods of determination, target sequences, and experimental conditions. Further research is needed to gain a better understanding of the kinetics (quantitative rate values) and *trans*-cleavage activities of Cas proteins.

Integrating a CRISPR–Cas system after nucleic acid amplification improves detection specificity. CRISPR–Cas systems recognize specific sequences of amplicons and differentiate them from byproducts of amplification reactions. The multiple turnover *trans*-cleavage activity of Cas12 and Cas13 results in repeated cleavage of nucleic acid signaling reporters, generating amplified readout signals for detection, thus improving sensitivity. Applications are exemplified by several CRISPR-mediated techniques for rapid and sensitive detection of SARS-CoV-2 (Table S1, ESI††).

Incorporating CRISPR–Cas before nucleic acid amplification enables enrichment of rare and low-abundance nucleic acid targets and depletion of unwanted abundant nucleic acids. The enrichment strategy uses the sequence-specific binding ability of dCas9 and dCas12 to specifically recognize dsDNA and ssDNA. The PAM-dependent cleavage by Cas9 depletes unwanted high-abundance nucleic acids.

The ability of CRISPR–Cas9 systems to unwind dsDNA to ssDNA at a moderate temperature (37 °C) makes CRISPR–Cas9 useful for developing isothermal exponential amplification techniques. Exponential amplification of nucleic acids requires separation of dsDNA to ssDNA after each round of amplification reactions. A special feature of Cas9 and nCas9 is their ability to unwind and/or nick dsDNA to generate ssDNA at 37 °C. Therefore, CRISPR–Cas systems eliminate the high temperature (*e.g.*, 95 °C) denaturation step commonly required for PCR.

A combination of CRISPR–Cas systems with functional nucleic acids (FNAs) and molecular translators enables the detection of non-nucleic acid targets, such as metal ions, proteins, and small drug molecules. FNAs and molecular translators convert the detection of non-amplifiable targets into surrogate nucleic acid targets, such as ssDNA, which activates CRISPR–Cas and generates amplified responses for highly sensitive detection.

The realization of CRISPR–Cas-based detection techniques is promising but still in its early stages. The use of CRISPR–Cas systems to detect non-nucleic acid targets with assistance of FNAs and molecular translators has only recently been explored and has incredible potential to be developed further. A broader application of CRISPR technology to non-nucleic acid targets can be achieved by incorporating other molecular translators.^97^

CRISPR–Cas systems have been used for DNA and RNA imaging in live cells,^40,41,93^ but the target site is limited to repetitive sequences, because of the low copy number of DNA or RNA in one cell and the lack of sufficient signal amplification,^98^ CRISPR–Cas-integrated amplification strategies allow for enhanced signal generation at a genomic locus in fixed cells and overcome the limitation of CRISPR–Cas probes that could only detect and image repetitive sequences.^42^ Amplification techniques using CRISPR–Cas systems have not been demonstrated to function within live cells. Perhaps a major reason is because *trans*-cleavage activity operates variably, if at all, within living cells. For example, *cis*-cleavage activity of Cas13 occurred in the modified human embryonic kidney cell line HEK293FT, while *trans*-cleavage activity was not observed.^43,99^ However, it has been reported that *trans*-cleavage activity occurred within LN229 glioma cells.^100^ The inconsistency may be attributed to the differences in cellular environments or methods of intracellular delivery from different studies.^100^ However, CRISPR–Cas-facilitated amplification strategies that do not depend on *trans*-cleavage activity may still work within live cells. The recently discovered Cas12f (Cas14) could be considered for use in live cells. Cas12f is only 400–700 amino acids long, which makes Cas12f easier to deliver into cells than other Cas effectors that are greater than 1000 amino acids in length. Due to the smaller size of Cas12f, both the Cas12f gene and sgRNA can be readily packaged^101^ in the same adeno-associated virus (AVV) vector and delivered into live cells.

RNases, nucleases, and proteases that degrade CRISPR–Cas ribonucleoproteins and nucleic acids are abundant in biological samples. These interferences need to be further addressed when CRISPR–Cas technologies are used as diagnostic tools.

CRISPR–Cas technologies continue to revolutionize analytical applications, and their full potential has yet to be reached. Tremendous advances made in modifications of Cas proteins and crRNA for the purpose of gene editing^102–106^ could be harnessed and applied to developing and improving molecular diagnostics.

## Author contributions

Wei Feng, Ashley M. Newbigging, Jeffrey Tao, Yiren Cao, Hanyong Peng, and Connie L, contributed to data curation, formal analysis, visualization, validation, writing – original draft, and writing – review & editing. Jinjun Wu and Bo Pang contributed to data curation, formal analysis, and writing a component of the original draft, review & editing. Juan Li, and D. Lorne Tyrrell, contributed to funding acquisition, resources, supervision, and writing – review & editing. Hongquan Zhang and X. Chris Le contributed to conceptualization, data curation, formal analysis, funding acquisition, project administration, resources, supervision, writing – original draft, and writing – review & editing. This feature review is a product of collaboration of four research groups led by Professors X. Chris Le, Juan Li, D. Lorne Tyrrell, and Hongquan Zhang. These four professors mentored their students and postdoctoral fellows who contributed jointly to this review paper.

## Conflicts of interest

There are no conflicts to declare.

## Abbreviations and terminology

Cas: “CRISPR-associated” proteins. Cas proteins work together with CRISPR RNA to target and cleave nucleic acids
*Cis*-cleavage activity: Target-initiated Cas activity that specifically cleaves the target nucleic acid
CRISPR: “Clustered regularly interspaced short palindromic repeats”. CRISPR sequences in prokaryotic genomes are fully expressed along with Cas proteins as a nuclease defense mechanism against viral infections
CRISPR–Cas: The nucleic acid targeting and cleaving system that involves CRISPR RNA, tracrRNA, or guide RNA, and Cas proteins
crRNA: “CRISPR RNA”. This is the sequence-specific targeting component of CRISPR–Cas systems that can be programmed for different target sequences
HEPN: Two “higher eukaryotes and prokaryotes nucleotide”-binding domains of Cas13 that mediate the cleavage of ssRNA
HNH: The domain of Cas9 protein that facilitates the cleavage of the target nucleic acid strand (the strand complementary to sgRNA)
Protospacer: The region of the target nucleic acid sequence that is complementary to the spacer region of sgRNA or crRNA
PAM: “Protospacer adjacent motif”. This sequence is located 3–4 nucleotides downstream of protospacer regions and is intimately involved in Cas activation
PAMmer: “PAM-presenting oligonucleotides” that hybridize with ssDNA or ssRNA to stimulate the site-specific nuclease activity of Cas9
PFS: “Protospacer flanking site”. Cas13 requires only a single nucleotide downstream of the protospacer region instead of a complete PAM region
RuvC: The domain that facilitates Cas9 cleavage activity of the non-target nucleic acid strand (the strand in dsDNA target that does not hybridize with crRNA/sgRNA), and Cas12 cleavage activity
sgRNA: “Single-guide RNA”. This RNA combines both crRNA and tracrRNA to form one RNA that dually functions to anchor Cas proteins and bind to target nucleic acids
SNP: “Single-nucleotide polymorphism”, variation of a specific genome site among individuals of the same species. Some SNPs are associated with the susceptibility to diseases
Spacer: The region of crRNA or sgRNA that is complementary to the target nucleic acid sequence
tracrRNA: “*Trans*-activating CRISPR RNA”. This RNA is hybridized with crRNA and functions to bind Cas proteins
*Trans*-cleavage activity: Target-initiated Cas activity that non-specifically cleaves nucleic acid

## Supplementary Material

SC-012-D0SC06973F-s001
