# Supplementary material for: CRISPR technology incorporating amplification strategies: molecular assays for nucleic acids, proteins, and small molecules
Source: Chem Sci. 2021 Mar 2;12(13):4683–98. doi: 10.1039/d0sc06973f (PMC8179559; doi:10.1039/d0sc06973f)
Supplement: SC-012-D0SC06973F-s001 [file SC-012-D0SC06973F-s001.pdf]

Electronic Supplementary Material (ESI) for Chemical Science.
This journal is © The Royal Society of Chemistry 2021

Electronic supplementary information (ESI)

CRISPR Technology Incorporating Amplification Strategies: Molecular Assays for Nucleic Acids, Proteins, and Small Molecules

Wei Feng,[1,§] Ashley M. Newbigging,[1,§] Jeffrey Tao,[1,§] Yiren Cao,[1,§] Hanyong Peng,[1,§,+] Connie Le,[2,§] Jinjun Wu,[1] Bo Pang,[1,3] Juan Li,[3] D. Lorne Tyrrell,[2] Hongquan Zhang,[1,*] X. Chris Le [1,*]

1. Division of Analytical and Environmental Toxicology, Department of Laboratory Medicine and Pathology, Faculty of Medicine and Dentistry, University of Alberta, Edmonton, Alberta, Canada T6G 2G3

2. Li Ka Shing Institute of Virology, Department of Medical Microbiology and Immunology, Faculty of Medicine and Dentistry, University of Alberta, Edmonton, Alberta, Canada T6G 2E1

3. School of Public Health, Jilin University, 1163 Xinmin Street, Changchun, Jilin, China 130021

*Corresponding authors.
Telephone: 1-780-492-6416
Fax: 1-780-492-7800
E-mail: xc.le@ualberta.ca, hongquan@ualberta.ca

[§] These authors contributed equally.

+ Present address: State Key Laboratory of Environmental Chemistry and Ecotoxicology, Research Center for Eco-Environmental Sciences, Chinese Academy of Sciences, Beijing, China 100085

Table of Content

Table S1. A summary of reported assays using CRISPR technology and amplification strategies for the detection of SARS-CoV-2
Figure S1. Amplification and calibration curves of an assay that integrates CRISPR-Cas within EXPAR for isothermal amplification.
Figure S2. Amplification and calibration curves of an assay that uses the *trans*-cleavage activity of CRISPR-Cas12a for the detection of small molecules.

Table S1. A summary of reported assays using CRISPR technology and amplification strategies for the detection of SARS-CoV-2

	Cas variants	Incorporated technique(s)	Readout	LOD	Specificity	Operation time	Ref.
1	LbaCas12a	RT-RPA & Gold nanoparticles	Colorimetric	1 copy/ reaction	High (N and ORF1ab gene)	~60 min*	1
2	LbaCas12a	RT-RPA	Fluorescence	6.25 copies/ µL	High (N gene)	~60 min*	2
3	LbaCas12a	RT-RPA	Fluorescence anisotropy	3 copies/ µL	High (N gene)	20 min*	3
4	Cas13	ligation-triggered transcription & light-up RNA aptmer	Fluorescence	82 copies/ reaction	High (N and S gene)	> 50 min*	4
5	LbuCas13	Droplet microfluidics	Fluorescence	6 copies/ µL	High (N gene)	> 60 min*	5
6	LbaCas12a	RT-RAA	Glucose meter	10 copies/ µL	High (N gene)	~60 min*	6
7	LbaCas12a	RT-RPA	Fluorescence	1 copy/ µL	High (N and ORF1ab gene)	50 min*	7
8	LbaCas12a	RT-RPA & microfluidic chip	Fluorescence & Smartphone	0.38 copies/ µL	High (N and ORF1ab gene)	15 min	8
9	LbuCas13	N/A	Fluorescence & Smartphone	~100 copies/ µL	High (N gene)	35 min	9
10	SpCas9	RT-RPA & Lateral Flow Assay	Lateral Flow Strip	100 copies/ reaction	High (ORF1ab gene)<br>Low (E gene)	38 min*	10
11	dCas9	Horseradish peroxidase	Colorimetric	140 pM	High (N gene)	100 min	11
12	LbaCas12a	RT-LAMP	Fluorescence	30-45 copies/ µL	High (N gene)<br>Low (E gene)	40 min*	12

13	LwaCas13a	RT-RPA & T7 transcription	Fluorescence & Lateral Flow Strip	10-100 copies/ µL	High (ORF1ab gene)	50 min	13
14	LbaCas12a	RT-LAMP & microfluidic chip & isotachophoresis	Fluorescence	10 copies/ µL	High (ORF1ab gene)<br>Low (E gene)	35 min	14
15	LbaCas12a	RT-LAMP	Fluorescence	5 copies/ µL	High (S gene)	45 min*	15
16	LbaCas12a	RT-LAMP & Modified crRNA	Fluorescence	3-300 copies/ reaction	High (N gene)	~40 min*	16
17	LbaCas12a	RT-LAMP	Fluorescence	20 copies/ reaction	High (ORF gene)	40 min*	17
18	LbaCas12a	RT-RPA	Fluorescence	< 10 copies/ reaction	High (N gene)	20 min*	18
19	AapCas12b	RT-LAMP	Fluorescence & Smartphone	0.033 copies/ µL	High (N gene)	60-95 min	19
20	AaCas12b	RT-RAA	Fluorescence	10 copies/ µL	High (Rdrp gene)	~60 min*	20
21	LbaCas12a	RT-LAMP	Fluorescence & Lateral Flow Strip	10 copies/ µL	High (N gene)<br>Low (E gene)	~40 min*	21

RT-RPA, Reverse Transcription Recombinase Polymerase Amplification; RT-RAA, Reverse Transcription Recombinase-Aided Amplification; RT-LAMP, Reverse Transcription Loop-mediated Isothermal Amplification.

*Not including nucleic acid extraction time

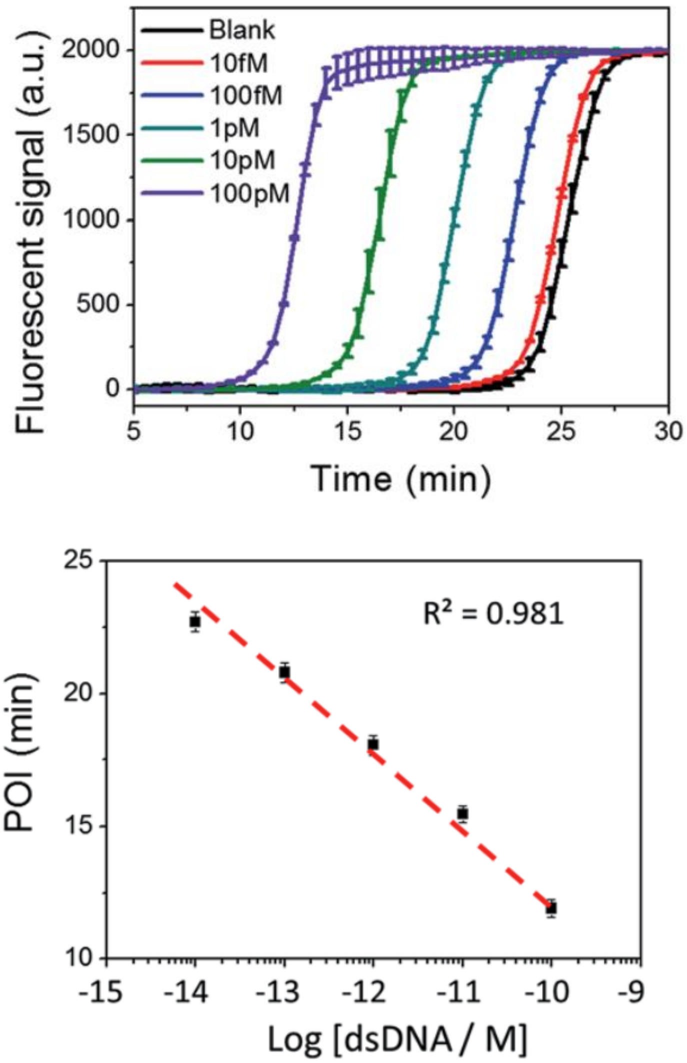

Figure S1. Amplification and calibration curves of an assay that integrates CRISPR-Cas within EXPAR for isothermal amplification.[22] (Ref. 75 in the main manuscript). POI: Point of inflection. Reproduced from reference 22 with permission from The Royal Society of Chemistry.

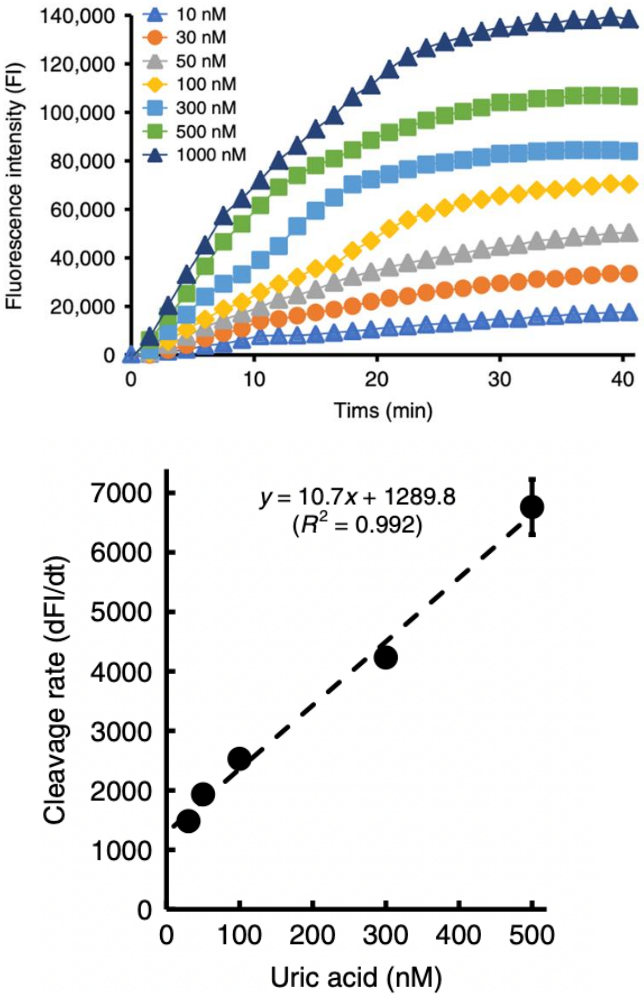

Figure S2. Amplification and calibration curves of an assay that uses the *trans*-cleavage activity of CRISPR-Cas12a for the detection of small molecules.[23] (Ref. 81 in the main manuscript). Reproduced with permission from reference 23. Copyright 2016 Springer Nature.

References:

1. Zhang WS, Pan J, Li F, Zhu M, Xu M, Zhu H, Yu Y, Su G. **Reverse Transcription Recombinase Polymerase Amplification Coupled with CRISPR-Cas12a for Facile and Highly Sensitive Colorimetric SARS-CoV-2 Detection**. Anal Chem. 2021 Feb 11. doi: 10.1021/acs.analchem.1c00013. Epub ahead of print. PMID: 33570401.

2. Samacoits A, Nimsamer P, Mayuramart O, Chantaravisoot N, Sitthi-Amorn P, Nakhakes C, Luangkamchorn L, Tongcham P, Zahm U, Suphanpayak S, Padungwattanachoke N, Leelarthaphin N, Huayhongthong H, Pisitkun T, Payungporn S, Hannanta-Anan P. **Machine Learning-Driven and Smartphone-Based Fluorescence Detection for CRISPR Diagnostic of SARS-CoV-2**. ACS Omega. 2021 Jan 20;6(4):2727-2733. doi: 10.1021/acsomega.0c04929. PMID: 33553890; PMCID: PMC7839157.

3. Lee CY, Degani I, Cheong J, Lee JH, Choi HJ, Cheon J, Lee H. **Fluorescence polarization system for rapid COVID-19 diagnosis**. Biosens Bioelectron. 2021 Jan 30;178:113049. doi: 10.1016/j.bios.2021.113049. Epub ahead of print. PMID: 33540323; PMCID: PMC7846465.

4. Wang Y, Zhang Y, Chen J, Wang M, Zhang T, Luo W, Li Y, Wu Y, Zeng B, Zhang K, Deng R, Li W. **Detection of SARS-CoV-2 and Its Mutated Variants via CRISPR-Cas13-Based Transcription Amplification**. Anal Chem. 2021 Jan 29:acs.analchem.0c04303. doi: 10.1021/acs.analchem.0c04303. Epub ahead of print. PMID: 33511840; PMCID: PMC7860141.

5. Tian T, Shu B, Jiang Y, Ye M, Liu L, Guo Z, Han Z, Wang Z, Zhou X. **An Ultralocalized Cas13a Assay Enables Universal and Nucleic Acid Amplification-Free Single-Molecule RNA Diagnostics.** ACS Nano. 2021 Jan 26;15(1):1167-1178. doi: 10.1021/acsnano.0c08165. Epub 2020 Dec 17. PMID: 33498106.

6. Huang D, Shi Z, Qian J, Bi K, Fang M, Xu Z. **A CRISPR-Cas12a-derived biosensor enabling portable personal glucose meter readout for quantitative detection of SARS-CoV-2.** Biotechnol Bioeng. 2021 Jan 6. doi: 10.1002/bit.27673. Epub ahead of print. PMID: 33410130.

7. Xiong D, Dai W, Gong J, Li G, Liu N, Wu W, Pan J, Chen C, Jiao Y, Deng H, Ye J, Zhang X, Huang H, Li Q, Xue L, Zhang X, Tang G. **Rapid detection of SARS-CoV-2 with CRISPR-Cas12a.** PLoS Biol. 2020 Dec 15;18(12):e3000978. doi: 10.1371/journal.pbio.3000978. PMID: 33320883; PMCID: PMC7737895.

8. Ning B, Yu T, Zhang S, Huang Z, Tian D, Lin Z, Niu A, Golden N, Hensley K, Threeton B, Lyon CJ, Yin XM, Roy CJ, Saba NS, Rappaport J, Wei Q, Hu TY. **A smartphone-read ultrasensitive and quantitative saliva test for COVID-19**. Sci Adv. 2021 Jan 8;7(2):eabe3703. doi: 10.1126/sciadv.abe3703. PMID: 33310733; PMCID: PMC7793573.

9. Fozouni P, Son S, Díaz de León Derby M, Knott GJ, Gray CN, D'Ambrosio MV, Zhao C, Switz NA, Kumar GR, Stephens SI, Boehm D, Tsou CL, Shu J, Bhuiya A, Armstrong M, Harris AR, Chen PY, Osterloh JM, Meyer-Franke A, Joehnk B, Walcott K, Sil A, Langelier C, Pollard KS, Crawford ED, Puschnik AS, Phelps M, Kistler A, DeRisi JL, Doudna JA, Fletcher DA, Ott M. **Amplification-free detection of SARS-CoV-2 with CRISPR-Cas13a**

and mobile phone microscopy. Cell. 2021 Jan 21;184(2):323-333.e9. doi: 10.1016/j.cell.2020.12.001. Epub 2020 Dec 4. PMID: 33306959; PMCID: PMC7834310.

10. Xiong E, Jiang L, Tian T, Hu M, Yue H, Huang M, Lin W, Jiang Y, Zhu D, Zhou X. **Simultaneous Dual-Gene Diagnosis of SARS-CoV-2 Based on CRISPR/Cas9-Mediated Lateral Flow Assay**. Angew Chem Int Ed Engl. 2020 Dec 9. doi: 10.1002/anie.202014506. Epub ahead of print. PMID: 33295064.

11. Moon J, Kwon HJ, Yong D, Lee IC, Kim H, Kang H, Lim EK, Lee KS, Jung J, Park HG, Kang T. **Colorimetric Detection of SARS-CoV-2 and Drug-Resistant pH1N1 Using CRISPR/dCas9**. ACS Sens. 2020 Dec 24;5(12):4017-4026. doi: 10.1021/acssensors.0c01929. Epub 2020 Dec 3. PMID: 33270431; PMCID: PMC7724983.

12. Pang B, Xu J, Liu Y, Peng H, Feng W, Cao Y, Wu J, Xiao H, Pabbaraju K, Tipples G, Joyce MA, Saffran HA, Tyrrell DL, Zhang H, Le XC. **Isothermal Amplification and Ambient Visualization in a Single Tube for the Detection of SARS-CoV-2 Using Loop-Mediated Amplification and CRISPR Technology.** Anal Chem. 2020 Dec 15;92(24):16204-16212. doi: 10.1021/acs.analchem.0c04047. Epub 2020 Nov 26. PMID: 33238709; PMCID: PMC7724759.

13. Arizti-Sanz J, Freije CA, Stanton AC, Petros BA, Boehm CK, Siddiqui S, Shaw BM, Adams G, Kosoko-Thoroddsen TF, Kemball ME, Uwanibe JN, Ajogbasile FV, Eromon PE, Gross R, Wronka L, Caviness K, Hensley LE, Bergman NH, MacInnis BL, Happi CT, Lemieux JE, Sabeti PC, Myhrvold C**. Streamlined inactivation, amplification, and Cas13-based detection of SARS-CoV-2.** Nat Commun. 2020 Nov 20;11(1):5921. doi: 10.1038/s41467-020-19097-x. PMID: 33219225; PMCID: PMC7680145.

14. Ramachandran A, Huyke DA, Sharma E, Sahoo MK, Huang C, Banaei N, Pinsky BA, Santiago JG. **Electric field-driven microfluidics for rapid CRISPR-based diagnostics and its application to detection of SARS-CoV-2.** Proc Natl Acad Sci U S A. 2020 Nov 24;117(47):29518-29525. doi: 10.1073/pnas.2010254117. Epub 2020 Nov 4. PMID: 33148808; PMCID: PMC7703567.

15. Wang R, Qian C, Pang Y, Li M, Yang Y, Ma H, Zhao M, Qian F, Yu H, Liu Z, Ni T, Zheng Y, Wang Y. **opvCRISPR: One-pot visual RT-LAMP-CRISPR platform for SARS-cov-2 detection**. Biosens Bioelectron. 2021 Jan 15;172:112766. doi: 10.1016/j.bios.2020.112766. Epub 2020 Oct 26. PMID: 33126177; PMCID: PMC7586109.

16. Nguyen LT, Smith BM, Jain PK. **Enhancement of trans-cleavage activity of Cas12a with engineered crRNA enables amplified nucleic acid detection.** Nat Commun. 2020 Sep 30;11(1):4906. doi: 10.1038/s41467-020-18615-1. Erratum in: Nat Commun. 2020 Nov 24;11(1):6104. PMID: 32999292; PMCID: PMC7528031.

17. Chen Y, Shi Y, Chen Y, Yang Z, Wu H, Zhou Z, Li J, Ping J, He L, Shen H, Chen Z, Wu J, Yu Y, Zhang Y, Chen H. **Contamination-free visual detection of SARS-CoV-2 with CRISPR/Cas12a: A promising method in the point-of-care detection.** Biosens Bioelectron. 2020 Dec 1;169:112642. doi: 10.1016/j.bios.2020.112642. Epub 2020 Sep 20. PMID: 32979593; PMCID: PMC7502227.

18. Ding X, Yin K, Li Z, Lalla RV, Ballesteros E, Sfeir MM, Liu C. **Ultrasensitive and visual detection of SARS-CoV-2 using all-in-one dual CRISPR-Cas12a assay.** Nat Commun. 2020 Sep 18;11(1):4711. doi: 10.1038/s41467-020-18575-6. PMID: 32948757; PMCID: PMC7501862.

19. Joung J, Ladha A, Saito M, Kim NG, Woolley AE, Segel M, Barretto RPJ, Ranu A, Macrae RK, Faure G, Ioannidi EI, Krajeski RN, Bruneau R, Huang MW, Yu XG, Li JZ, Walker BD, Hung DT, Greninger AL, Jerome KR, Gootenberg JS, Abudayyeh OO, Zhang F. **Detection of SARS-CoV-2 with SHERLOCK One-Pot Testing**. N Engl J Med. 2020 Oct 8;383(15):1492-1494. doi: 10.1056/NEJMc2026172. Epub 2020 Sep 16. PMID: 32937062; PMCID: PMC7510942.

20. Guo L, Sun X, Wang X, Liang C, Jiang H, Gao Q, Dai M, Qu B, Fang S, Mao Y, Chen Y, Feng G, Gu Q, Wang RR, Zhou Q, Li W. **SARS-CoV-2 detection with CRISPR diagnostics**. Cell Discov. 2020 May 19;6:34. doi: 10.1038/s41421-020-0174-y. PMID: 32435508; PMCID: PMC7235268.

21. Broughton JP, Deng X, Yu G, Fasching CL, Servellita V, Singh J, Miao X, Streithorst JA, Granados A, Sotomayor-Gonzalez A, Zorn K, Gopez A, Hsu E, Gu W, Miller S, Pan CY, Guevara H, Wadford DA, Chen JS, Chiu CY. **CRISPR-Cas12-based detection of SARS-CoV-2**. Nat Biotechnol. 2020 Jul;38(7):870-874. doi: 10.1038/s41587-020-0513-4. Epub 2020 Apr 16. PMID: 32300245.

22. Zhang K, Deng R, Li Y, Zhang L, Li J. **Cas9 cleavage assay for pre-screening of sgRNAs using nicking triggered isothermal amplification.** Chem Sci. 2016 Aug 1;7(8):4951-4957. doi: 10.1039/c6sc01355d. Epub 2016 Apr 29. PMID: 30155144; PMCID: PMC6018437.

23. Liang M, Li Z, Wang W, Liu J, Liu L, Zhu G, Karthik L, Wang M, Wang KF, Wang Z, Yu J, Shuai Y, Yu J, Zhang L, Yang Z, Li C, Zhang Q, Shi T, Zhou L, Xie F, Dai H, Liu X, Zhang J, Liu G, Zhuo Y, Zhang B, Liu C, Li S, Xia X, Tong Y, Liu Y, Alterovitz G, Tan GY, Zhang LX. **A CRISPR-Cas12a-derived biosensing platform for the highly sensitive detection of diverse small molecules.** Nat Commun. 2019 Aug 14;10(1):3672. doi: 10.1038/s41467-019-11648-1. PMID: 31413315; PMCID: PMC6694116.
